# Draft Genome Sequence of the Clover (*Trifolium repens* L.) Root Endophyte *Paraburkholderia* sp. Strain A27

**DOI:** 10.1128/genomeA.00466-17

**Published:** 2017-06-01

**Authors:** Aurelie Laugraud, Sandra Young, Emily Gerard, Maureen O’Callaghan, Steven Wakelin

**Affiliations:** a Lincoln Science Centre, AgResearch Ltd., Christchurch, New Zealand; b Scion Research, Christchurch, New Zealand

## Abstract

*Paraburkholderia* sp. strain A27, isolated from the root material of white clover, has plant growth-promoting activity on a range of agriculturally important plants. The draft genome of this bacterium is 7,393,089 bp and harbors a range of genes putatively involved in host colonization.

## GENOME ANNOUNCEMENT

The betaproteobacterial genus *Paraburkholderia* encompasses a range of environmentally associated species, many of which were recently reclassified from *Burkholderia* (1). Several species have been found to live in close association with plant tissues, possibly as endophytes, and they may protect plants from disease or increase plant growth through biofertilizer or other means (2–4). As such, the discovery of *Paraburkholderia* strains with such traits is of considerable agronomic importance (e.g., 5, 6). During a study to discover root-associated bacteria with plant growth-promoting (PGP) activity (7), we isolated *Paraburkholderia* sp. strain A27 from the washed roots of *Trifolium repens* (cv. Huia) grown in pallic soil (8) collected from Waipapa, New Zealand. The strain was able to mineralize phytate and solubilize mineral phosphate, and it had no *in vitro* antifungal activity (7) but exhibited strong PGP activity in glasshouse experiments. Based on the strain’s 16S rRNA sequence, the closest phylogenetic association is to *Paraburkholderia terricola* (GenBank accession no. NR_029044); however, further and formal identification of strain A27 has not been conducted. In order to unravel the mechanisms of PGP activity, the genome of this strain was sequenced.

Genomic DNA was extracted using the ISOLATE II genomic DNA kit (Bioline, USA) and sequenced on a MiSeq platform (Illumina). Libraries were prepared by sonication with Covaris or Bioruptor to produce fragments of 500 bp in size, extended by Klenow DNA polymerase, and ligated into T-vector. We used two libraries to sequence this genome; the first had an insert of 450 bp (paired-end), and the second 8,000 bp (mate pair). Sequencing produced 18 million (~70-fold coverage) reads of 120 bp each. The fragments were assembled in A5-miseq (9), with default settings, and SSPACE (10). The final assembly has 28 scaffolds, with an *N*_50_ of 2,132,757 bp. The genome was annotated using the NCBI Prokaryotic Genome Annotation Pipeline (11).

The draft genome of *Paraburkholderia* A27 is 7,393,089 bp and has a G+C content of 65.4%. A total of 6,736 genes are annotated, encoding 6,673 proteins. The genome has 54 tRNAs and no predicted clustered regularly interspaced short palindromic repeat (CRISPR) clusters, and it harbors numerous phage-associated genes (e.g., virion morphogenesis, phage portal, tail and tail measure, and head decoration proteins). The genome is predicted to have *nodJ* and *nodI* nodulation genes and a nodulation factor ABC transporter ATP-binding protein. Given the isolation of this strain from a legume host and the ability for some *Paraburkholderia* spp. to form nodules on legumes (2), this may have functional significance for the strain; however, this has not been experimentally validated. While the phenazine biosynthesis gene *phzF* was present, other antifungal genes were absent, and the strain has not exhibited *in vitro* antifungal activity (7). As such, the *phzF* gene may have a role in host-plant colonization (virulence) rather than formation of antifungal compounds in this strain. The genome also possesses 5 siderophore-associated genes (TonB-dependent siderophore receptor and NADPH-dependent ferric siderophore reductase) (10). However, clear genetic leads to the basis of the PGP by this strain remain elusive and may reside in one of the 25% predicted proteins which remain hypothetical.

### Accession number(s).

This whole-genome shotgun project has been deposited at DDBJ/ENA/GenBank under the accession no. MWJQ00000000. The version described in this paper is version MWJQ01000000.
